# Association of severity and prognosis with elevated blood pressure and heart rate levels in patients with intracerebral hemorrhage

**DOI:** 10.1186/s12883-023-03409-x

**Published:** 2023-10-06

**Authors:** Dandan Wang, Ruixuan Jiang, Kaijiang Kang, Anxin Wang, Xiaoli Zhang, Jingjing Lu, Xingquan Zhao

**Affiliations:** 1 Department of Neurology, Beijing Tiantan Hospital, Capital Medical University, No.119, South 4th Ring Road, Fengtai District, Beijing, 100070 China; 2 National Clinical Research Center for Neurological Diseases, Beijing, China; 3 Research Unit of Artificial Intelligence in Cerebrovascular Disease, Chinese Academy of Medical Sciences, Beijing, China

**Keywords:** Intracerebral hemorrhage, Blood pressure, Heart rate, Prognosis

## Abstract

**Background:**

Intracerebral hemorrhage (ICH) has a high mortality and morbidity in the world. Elevated blood pressure (BP) and heart rate (HR) have been identified as independent risk factors, with potential to predict prognosis and recurrence of cardiovascular diseases. Our study aimed to elucidate the association between BP and HR levels and the severity, as well as prognosis, of patients diagnosed with ICH.

**Methods:**

The basic characteristics of patients and laboratory examination results, inclusive of BP and HR levels upon admission, were recorded as baseline data. The modified Rankin Scale and living status were taken into account for all patients at a 1-year follow-up. The relationship between various BP and HR levels and clinical outcome was analyzed using logistic regression and the Kaplan-Meier survival method.

**Results:**

A total of 1,416 patients with acute ICH from 13 hospitals in Beijing were enrolled in our study. Logistic regression analysis indicated that patients with higher HR and BP (group 4), along with those with higher HR but lower BP (group 2), exhibited a poorer prognosis compared to those with lower BP and HR (group 1). This result was particularly pronounced in younger, male subgroups (OR (95% CI) = 4.379(2.946–6.508), P < 0.0001 for group 4; OR (95% CI) = 1.819 (1.219–2.714), P = 0.0034 for group 2). At the 1-year follow-up, group 4 patients demonstrated a significantly higher rate of fatal incidence compared to other groups (P < 0.01).

**Conclusions:**

Higher HR and BP levels, suggestive of an autonomic dysfunction, were independently associated with a poorer 1-year prognosis and reduced survival rate in ICH patients. Our findings underscore the need for early intervention to modulate these physiological parameters in patients with ICH.

## Introduction

Intracerebral hemorrhage (ICH) represents a grave subtype of stroke and constitutes the primary cause of death and disability within the spectrum of cerebrovascular diseases. The incidence of ICH is notably elevated in Asian countries, particularly in China [1–3]. ICH places patients under significant duress, leading to a poorer prognosis and a substantial economic burden. It is estimated that approximately 40.4% of ICH patients do not survive past the first 30 days post-incident, with only a mere 12–39% managing to attain functional independence six months after the event [3, 4]. Blood pressure (BP) and heart rate (HR) are fundamental vital signs that can be assessed during emergency ambulance transport or upon hospital admission, as necessary. Prior studies have indicated that fluctuations in blood pressure and heart rate, along with impaired baroreflex sensitivity (primarily indicative of autonomic dysregulation), are correlated with poor outcomes in ischemic stroke and other cardiovascular diseases [5, 6]. While previous research on autonomic function in the context of acute ICH is limited, our study aims to elucidate the relationship between blood pressure, heart rate, and the severity and prognosis of patients with ICH.

## Methods

### Study design and population

This study was a prospective, multicenter, consecutive observational cohort study, conducted in 13 hospitals in Beijing from January 2014 to September 2016. Patients of acute ICH were screened by researches. The inclusion criteria include: (1) ICH was diagnosis by the WHO standard and confirmed by the hospital’s computerized tomography (CT) scan [7]; (2) age ≥ 18 years old; (3) arriving at hospital within 72 h after onset; (4) first-ever acute-onset ICH; (5) written informed consent was obtained. The exclusion criteria include: (1) past history of ICH; (2) congenital or acquired coagulation disorders; (3) complicated with major comorbidities or late-stage diseases. A total of 1,964 patients were enrolled. We excluded 171 patients with missing blood pressure and heart rate records, 7 patients with invalid records of heart rate, and 370 patients without the complete follow-up records. Finally, 1,416 patients were enrolled in our study.

The study protocol conforms to the ethical guidelines of the 1975 Declaration of Helsinki, as reflected in a priori approval by the institution’s human research committee. The study was also approved by the Institutional Review Board (IRB) of Beijing Tiantan Hospital, Capital Medical University (No. KY2014-023-02). Written informed consents were obtained from all patients or their relatives. All the centers were given a unified and standard training about the questionnaire collection, testing methods of laboratory indexes and the interpretation of ICH at the beginning of the study, and regular inspections were given to ensure the quality of the study. All the images were collected to our study group and reanalyzed at the end of the study. The patients were also informed of abnormal findings and recommended treatments.

### Assessment of BP and HR

For BP measurements, patients were either seated or lying in a quiet setting prior to the procedure, which was performed using a mercury manometer. A 10-second, 12-lead electrocardiogram was conducted to determine the HR after the individual had rested in the supine position for 5 min. Both BP and HR were measured upon the patient’s first admission to the hospital.

We stratified the patients into four groups based on their BP and HR levels. Group 1 was characterized by lower BP (below the average systolic BP level identified in this study) and lower HR (below the average HR level in this study). Group 2 comprised individuals with lower BP (below the average systolic BP level in this study) and higher HR (above the average HR level in this study). Group 3 consisted of patients with higher BP (above the average systolic BP level in this study) and lower HR (below the average HR level in this study). Finally, group 4 was defined as having higher BP (above the average systolic BP level in this study) and higher HR (above the average HR level in this study).

### Assessment of epidemiological information and ICH relevant characteristics

Each patient completed a standardized questionnaire (including age, gender, ethnicity, and other basic information) administered by our trained investigators. Smoking was classified as consuming at least one cigarette per day for over a year. Drinking was defined as an intake of at least 80 g of liquor daily for more than one year. Cessation of smoking or drinking was only acknowledged if it had been sustained for at least one year. Body weight (accurate to 0.1 kg) and height (accurate to 0.1 cm) were measured, and the body mass index (BMI) was computed as body weight (kg) divided by the square of the height (m2). Pulmonary infection was diagnosed by treating physician according to the chest CT or X-ray after admission to hospital [8, 9]. The definitions of other comorbid diseases and laboratory examinations referenced in this study have been detailed in our previous articles [9].

The location, hematoma volume, and etiology of ICH were recorded during patients’ hospitalization. Etiology was categorized as hypertension, cerebral amyloid angiopathy (CAA), secondary, and others. The secondary etiology encompassed conditions like aneurysms, arteriovenous malformations, arteriovenous fistulas, cavernous hemangiomas, venous malformations, telangiectasia, venous sinus thrombosis, moyamoya disease, and coagulation disorders. ICH resulting from trauma or neoplasms was excluded from our study. We also evaluated the National Institute of Health Stroke Scale (NIHSS), Glasgow Coma Scale (GCS), and modified Rankin Scale (mRS) upon hospital admission and at discharge, as well as the mRS at 1-month, 3-month, and 1-year intervals during the follow-up period for each patient, conducted by our specifically trained doctors.

### Follow-up and outcome assessment

All patients were subjected to a face-to-face interview at discharge, followed by telephone interviews at 1-month, 3-month, and 1-year intervals post-ICH. Research coordinators, who were blinded to patients’ baseline characteristics, evaluated mRS scores based on the functional status reported by the patients, their relatives, or caregivers during each follow-up. A poor functional outcome or prognosis, implying death or disability, was characterized by a score of 3 to 6 on the mRS. A score of 6, indicating death, was meticulously documented with the date and etiology of death, and analyzed separately.

### Data management and statistical analysis

Data management was facilitated by the SAS software (version 9.3; SAS Institute, Cary, North Carolina, USA). All continuous variables underwent a normality test, all of which returned as non-normally distributed. Descriptive statistics comprised quartiles for continuous variables and percentages for categorical variables. The chi-squared test was employed to compare categorical variables, whereas the Kruskal-Wallis test was used for continuous variables. Logistic regression and the Kaplan-Meier survival method were applied to analyze the relationship between different BP and HR groups and clinical outcome. The null hypothesis was rejected for P < 0.05.

## Results

**Fig. 1:**
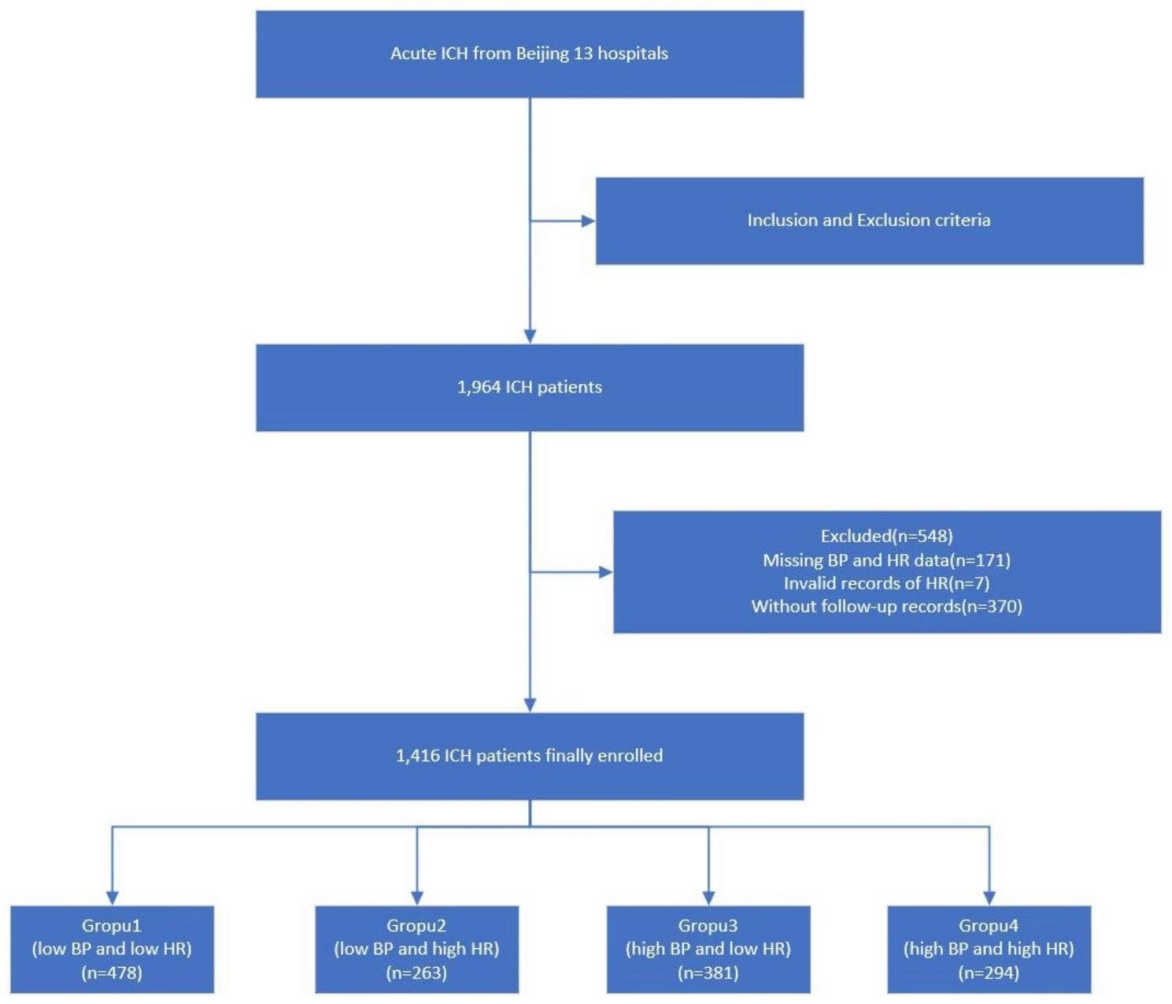
The flowchart of the study

A flowchart of this study is depicted in Fig. 1. From the cohort of 1,416 patients, 948 were males and 468 were females. The average age of the patients was 57.5 ± 14.3 years old. The details of systolic BP and HR for the whole and each of the four groups’ participants were described in Table 1. Table 2 outlines the baseline characteristics of the patients. Relative to patients with lower BP and HR, those with higher BP and HR exhibited a higher BMI level, glucose concentration, and incidence of hypertension and pulmonary infection. Smoking and alcohol consumption seemed to be more prevalent in the lower BP and HR group. The average prior mRS was 0.29, 0.38, 0.27, and 0.36 respectively, which also varied among the different BP and HR groups (all P values < 0.05). Other epidemiological characteristics, laboratory results, and comorbid diseases showed no significant difference among the groups (Table 2).

**Table 1:** Details of the systolic BP (mmHg) and HR (/min) among groups

	minimum value	25% quartile	50% quartile	75% quartile	maximum value	mean	standard deviation
systolic BP:							
whole (n = 1,416)	82.0	147.0	164.0	186.0	280.0	166.8	29.7
group 1 (n = 478)	95.0	131.0	147.0	157.0	166.0	143.6	15.3
group 2 (n = 263)	82.0	135.0	149.0	158.0	166.0	145.0	15.8
group 3 (n = 381)	167.0	175.0	186.0	200.0	270.0	189.7	18.8
group 4 (n = 294)	167.0	178.0	190.0	208.0	280.0	194.5	21.2
HR:							
whole (n = 1,416)	38.0	70.0	79.0	87.5	170.0	80.9	16.0
group 1 (n = 478)	38.0	68.0	72.0	78.0	80.0	71.4	7.7
group 2 (n = 263)	81.0	84.0	90.0	100.0	170.0	94.1	14.0
group 3 (n = 381)	46.0	67.0	72.0	78.0	80.0	71.2	7.3
group 4 (n = 294)	81.0	85.0	93.5	105.0	169.0	97.0	14.6

BP: blood pressure, HR: heart rate

group 1: lower blood pressure and lower heart rate group; group 2: lower blood pressure and higher heart rate group; group 3: higher blood pressure and lower heart rate group; group 4: higher blood pressure and higher heart rate group

**Table 2:** Baseline characteristics and their univariate association with different blood pressure and heart rate levels

	Blood pressure and heart rate groups				P value
	group 1 n = 478	group 2 n = 263	group 3 n = 381	group 4 n = 294	
Male– no.(%)	324(67.8)	172(65.4)	247(64.8)	205(69.7)	0.5251
Age (IQR) -yr	59.0(49.0,68.0)	57.0(47.0,67.0)	57.0(49.0,66.0)	57.0(48.0,66.0)	0.4994
Ethnic = Han– no.(%)	443(92.7)	245(93.2)	356(93.4)	270(91.8)	0.6581
BMI (IQR)	24.5(22.0,27.0)	24.6(22.5,27.0)	25.4(23.1,27.9)	25.7(23.4,28.8)	0.0003
Smoking– no.(%)	161(33.7)	85(32.3)	125(32.8)	77(26.2)	0.0132
Drinking– no.(%)	192(40.2)	91(34.6)	131(34.4)	105(35.7)	0.0016
Previous mRS (IQR)	0(0,0)	0(0,0)	0(0,0)	0(0,0)	0.0325
FBG (IQR) - mmol/l	5.4(4.6,6.6)	5.7(4.9,7.3)	6.1(5.2,7.7)	6.7(5.4,9.3)	< 0.0001
HbA1c (IQR) -%	5.6(5.3,6.2)	5.6(5.2,6.4)	5.6(5.3,6.1)	5.7(5.3,6.6)	0.6415
TC (IQR) - mmol/l	4.5(3.8,5.1)	4.5(3.8,5.2)	4.6(4.0,5.3)	4.5(3.8,5.3)	0.1363
TG (IQR) - mmol/l	1.2(0.9,1.7)	1.2(0.9,1.8)	1.3(0.9,1.8)	1.3(0.8,1.7)	0.7402
LDL-C (IQR) - mmol/l	2.7(2.1,3.4)	2.6(2.1,3.3)	2.8(2.2,3.4)	2.7(2.2,3.3)	0.7599
Complicating disease– no.(%)					
Hypertension	262(54.8)	130(49.4)	267(70.1)	219(74.5)	< 0.0001
DM	58(12.1)	34(12.9)	46(12.1)	54(18.4)	0.0593
Dyslipidemia	105(22.0)	53(20.2)	68(17.9)	50(17.0)	0.2895
PI	99(20.7)	90(34.2)	106(27.8)	121(41.2)	< 0.0001
DVT	33(6.9)	16(6.1)	20(5.3)	22(7.5)	0.6467
AF	10(2.1)	6(2.3)	7(1.8)	5(1.7)	0.9577

IQR: interquartile range, BMI: Body Mass Index, mRS: modified Rankin Scale, FBG: fasting blood glucose, HbA1c: hemoglobin A1c, TC: total cholesterol, TG: triglyceride, LDL-C: low-density lipoprotein cholesterol, DM: Diabetes Mellitus, PI: Pulmonary infection, DVT: Deep vein thrombosis, AF: Atrial fibrillation

group 1: lower blood pressure and lower heart rate group; group 2: lower blood pressure and higher heart rate group; group 3: higher blood pressure and lower heart rate group; group 4: higher blood pressure and higher heart rate group

Next, we compared the characteristics of ICH among patients with different BP and HR levels. Relative to the lower BP and HR group, patients in the higher BP and HR group appeared to have a higher percentage of deep ICH location and hypertensive ICH. The volume of the hematoma was significantly larger in the higher BP and HR group compared to the lower BP and/or HR groups. Moreover, the percentage of ICH breaching into the ventricle and the number of patients who underwent surgical therapy were also higher in the higher BP and HR group (all P values < 0.05) (Table 3).

**Table 3:** Association with ICH characteristics among different blood pressure and heart rate levels

	Blood pressure and heart rate groups				P value
	group 1	group 2	group 3	group 4	
Location– no.(%)					0.0040
lobar	99(23.1)	61(25.1)	54(16.0)	33(12.8)	
deep	293(68.5)	151(62.1)	253(74.9)	195(75.6)	
ventricle	13(3.0)	11(4.5)	11(3.3)	8(3.1)	
lober + deep	23(5.4)	20(8.2)	20(5.9)	22(8.5)	
Etiology– no.(%)					< 0.0001
hypertension	328(68.7)	200(76.1)	343(90.0)	274(93.2)	
CAA	18(3.8)	8(3.0)	3(0.8)	0(0)	
secondary	68(14.2)	30(11.4)	16(4.2)	7(2.4)	
others	64(13.4)	25(9.5)	19(5.0)	13(4.4)	
hematoma volume (IQR)- ml	12.1(5.0,31.4)	15.4(6.8,37.0)	16.1(7.1,35.8)	23.6(8.2,52.2)	< 0.0001
break into ventricle– no.(%)	128(30.3)	106(45.5)	124(37.7)	144(56.9)	< 0.0001
break into subarachnoid– no.(%)	65(15.4)	43(18.6)	41(12.6)	57(22.9)	0.0075
Surgery– no.(%)	69(14.4)	48(18.3)	83(21.8)	84(28.6)	< 0.0001

IQR: interquartile range, CAA: Cerebral amyloid angiopathy

**Fig. 2:**
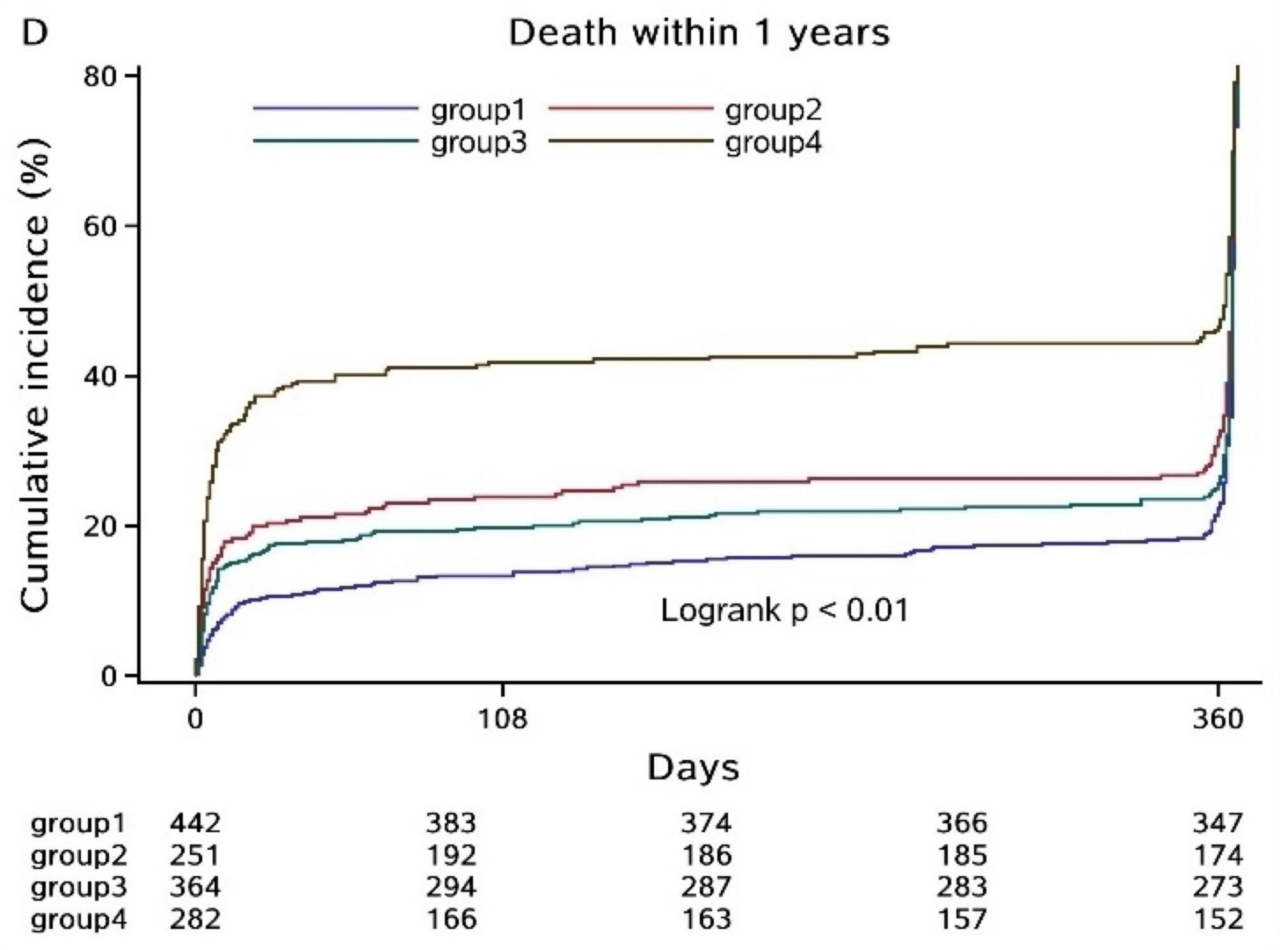
Kaplan-Meier curve of different blood pressure and heart rate groups for survival in one year group 1: lower blood pressure and lower heart rate group; group 2: lower blood pressure and higher heart rate group; group 3: higher blood pressure and lower heart rate group; group 4: higher blood pressure and higher heart rate group

In the third phase, we primarily investigated the association between BP and HR levels and the severity and prognosis of ICH patients. Regarding ICH severity, the higher BP and HR group demonstrated significantly lower GCS scores and higher NIHSS during hospitalization. Incidences of death were also higher in the higher BP and HR group. At the 1-month, 3-month, and 1-year follow-up intervals, groups 2, 3, and 4, particularly group 4, exhibited a significantly poorer functional prognosis and a higher mortality rate than group 1 (P < 0.05, Table 4). In the logistic regression analysis, higher BP and HR was identified as an independent risk factor for 1-year poor prognosis prevalence. After adjusting for relevant basic epidemiological factors (gender, age, ethnicity, BMI, smoking, drinking, prior mRS), the differences remained significant in group 2 [Odd Ratio (OR) (95% confidence interval (CI)) = 1.819 (1.219–2.714), P = 0.0034], and group 4 [OR (95% CI) = 4.379 (2.946–6.508), P < 0.0001] compared to group 1, respectively. In subgroup analysis, group 4 had a significantly poorer prognosis than group 1 across all gender and age subgroups (P < 0.05). However, group 2 only demonstrated a poorer prognosis than group 1 in male and younger ( < = 60 years old) subgroups (P < 0.05). Group 3 showed a trend towards a poorer prognosis in the female subgroup, but the difference did not reach statistical significance (P = 0.0568) (Table 5). During the 1-year follow-up period, we recorded the date of death for any patient who died due to any ICH-related or unrelated cause, and defined a cumulative incidence as the total mortality rate. A Kaplan-Meier survival curve for different BP and HR levels was plotted. Patients with higher BP and HR had a significantly higher rate of cumulative incidence compared to those with lower BP and HR (P < 0.01) (Fig. 2).

**Table 4:** Association with ICH severity and prognosis among different blood pressure and heart rate levels

	Blood pressure and heart rate groups				P value
	group 1	group 2	group 3	group 4	
GCS at first admission (IQR)	14.0(12.0,15.0)	14.0(7.0,15.0)	14.0(9.0,15.0)	10.0(5.0,14.0)	< 0.0001
NIHSS at first admission (IQR)	7.0(2.0,14.00)	10.0(4.0,24.0)	10.0(4.0,20.0)	17.5(9.0,28.0)	< 0.0001
GCS at discharge (IQR)	15.0(14.0,15.0)	15.0(13.0,15.0)	15.0(13.0,15.0)	15.0(10.0,15.0)	< 0.0001
NIHSS at discharge (IQR)	4.0(0.5,10.0)	7.0(2.0,15.0)	6.0(1.5,12.0)	8.0(3.0,18.5)	< 0.0001
Death within hospital– no.(%)	30(6.3)	28(10.7)	50(13.1)	85(28.9)	< 0.0001
mRS at discharge (IQR)	3.0(1.0,4.0)	4.0(1.0,5.0)	4.0(1.0,4.0)	4.0(3.0,6.0)	< 0.0001
Poor prognosis at discharge– no.(%)	240(50.2)	165(62.7)	238(62.5)	224(76.2)	< 0.0001
mRS at 1- month (IQR)	2.0(1.0,4.0)	4.0(2.0,5.0)	4.0(1.0,5.0)	4.0(3.0,6.0)	< 0.0001
Poor prognosis at 1- month– no.(%)	237(49.6)	169(64.3)	230(60.5)	225(76.5)	< 0.0001
Death at 1- month– no.(%)	50(10.5)	51(19.4)	65(17.1)	112(38.1)	< 0.0001
mRS at 3- months (IQR)	2.0(1.0,4.0)	3.0(1.0,5.0)	3.0(1.0,5.0)	4.0(2.0,6.0)	< 0.0001
Poor prognosis at 3- months– no.(%)	197(41.2)	154(58.8)	210(55.1)	212(72.1)	< 0.0001
Death at 3- months– no.(%)	59(12.3)	55(20.9)	69(18.1)	118(40.1)	< 0.0001
mRS at 1- year (IQR)	2.0(1.0,3.0)	3.0(1.0,5.0)	2.0(1.0,5.0)	4.0(2.0,6.0)	< 0.0001
Poor prognosis at 1- year– no.(%)	174(36.4)	142(54.0)	183(48.0)	196(66.7)	< 0.0001
Death at 1- year– no.(%)	80(16.7)	65(24.7)	86(22.6)	127(43.2)	< 0.0001

IQR: interquartile range, GCS: Glasgow Coma Scale, NIHSS: National Institute of Health Stroke Scale, mRS: modified Rankin Scale

**Table 5:** Odd ratios of blood pressure and heart rate levels for the presence of poor prognosis at 1- year

	OR	95% CI	P value
Crude			
group 1	Reference	Reference	Reference
group 2	2.050	1.510–2.784	< 0.0001
group 3	1.615	1.228–2.124	0.0006
group 4	3.494	2.573–4.743	< 0.0001
Adjusted1			
group 1	Reference	Reference	Reference
group 2	1.819	1.219–2.714	0.0034
group 3	1.414	0.997–2.006	0.0522
group 4	4.379	2.946–6.508	< 0.0001
Subgroup of gender			
Male adjusted2			
group 1	Reference	Reference	Reference
group 2	1.984	1.203–3.273	0.0073
group 3	1.192	0.774–1.836	0.4254
group 4	4.918	3.059–7.906	< 0.0001
Female adjusted2			
group 1	Reference	Reference	Reference
group 2	1.501	0.744–3.031	0.2567
group 3	1.851	0.982–3.486	0.0568
group 4	3.178	1.517–6.659	0.0022
Subgroup of age			
> 60 years old adjusted3			
group 1	Reference	Reference	Reference
group 2	1.629	0.850–3.121	0.1416
group 3	1.534	0.880–2.672	0.1310
group 4	3.817	1.947–7.483	< 0.0001
<=60 years old adjusted3			
group 1	Reference	Reference	Reference
group 2	1.797	1.081–2.987	0.0237
group 3	1.472	0.929–2.334	0.1001
group 4	4.539	2.772–7.432	< 0.0001

Adjusted1: adjusted by gender, age, ethnic, BMI, smoking, drinking, previous mRS

Adjusted2: adjusted by age, ethnic, BMI, smoking, drinking, previous mRS

Adjusted3: adjusted by gender, ethnic, BMI, smoking, drinking, previous mRS

## Discussion

The present study demonstrated that higher levels of blood pressure (BP) and heart rate (HR) are independently associated with a poorer prognosis in patients with acute intracerebral hemorrhage (ICH), especially among younger males. A Kaplan-Meier survival curve revealed that elevated BP and HR levels are also correlated with a decreased survival rate within a year following an ICH incident. Among these results, HR may play a more significant role than BP in the determination of functional prognosis and mortality.

Hypertension is widely recognized as the primary cause of ICH. However, approximately 75% of acute stroke patients experience elevated BP levels (referring to post-stroke BP) — a figure higher than that seen in other acute illnesses [10, 11]. A higher BP is usually found when ICH patients admitted to the hospital [12–14]. This suggests that the variability and increase in BP may not only be a cause of stroke, but also a result of the stroke event. Actually, HR is also an important index in many diseases. Previous studies have demonstrated that faster HR could predict morbidity and functional prognosis in a variety of cardiovascular diseases [15], including ischemic stroke [16–18], but few researchers focus on the hemorrhagic event. Only a post hoc pooled analysis of INTERACT reported a higher admission heart rate is independently associated with death and poor functional outcome after acute ICH [19]. Therefore, in our study, we integrated these two vital signs to assess their impact on ICH condition and prognosis. Our findings indicate that both higher BP and HR have a negative impact on the volume of the hematoma, severity, functional prognosis, and mortality rate in acute ICH cases. Even patients with relatively lower BP but higher HR experienced a poorer prognosis. Therefore, the pathogenesis and influences of BP and HR variations at the onset of ICH warrant further discussion.

Patients with ICH often exhibit higher BP than their pre-ICH levels, a phenomenon referred to as a hypertensive response to ICH [20]. Although initial BP and HR might spontaneously decrease over subsequent days, the adverse effects persist, leading to a poor prognosis for these patients. This is potentially due to disturbed autoregulation, damage to the autonomic nervous system, or as a consequence of headache and other neurological impairments [10, 20]. Similar to BP, HR variability provides significant insights into the autonomic nervous system. An increasing resting HR could elevate the BP level via autonomic regulation [21]. Both elevated BP and HR are the markers of increased sympathoadrenal tone [19] and the predictive factors in detection of autonomic impairment and neurological disorders [22]. It’s hypothesized that the initial hemorrhage attack may damage or compress brain regions that mediate autonomic control of BP and HR. Tang and Tian’s used BP variability, HR variability, and baroreflex sensitivity to present autonomic function and found out the autonomic dysfunction portend an unfavorable outcome and prognosis of stroke patients [5, 23]. In our study, we discovered that, irrespective of BP levels, patients with higher HR had a poorer prognosis than those with lower BP and HR. This underlines the detrimental impact of autonomic dysfunction in ICH.

Interestingly, we found that in younger and male patients, higher HR may independently predict poor ICH prognosis, regardless of BP levels. Different age and gender groups may exhibit different capacities to regulate autonomic function. As a person ages, their maximal HR decreases, and this seems to be the result of changes in both intrinsic heart rate and chronotropic β-adrenergic responsiveness [24–26]. When a hemorrhagic attack occurs, the autonomic function of younger patients is immediately interrupted and disturbed, leading to a larger impact of HR on ICH in younger patients compared to older ones. For different gender participants, since women always had a less favorable prognosis of stroke than men, and their HR are consistently higher compared with men [24, 27]. We assumed the male ICH patients made a more susceptible to the pathogenic processes of autonomic dysfunction and present a more obviously variable on HR than women.

Potential limitations of our study should be discussed. First, the participants were all from Beijing, northern China, and so lifestyle or genetic factors specific to this region may influence BP and HR levels, leading to potential bias. Second, we did not monitor BP and HR levels in the days following the onset of ICH, meaning that we could not analyze the variability of these measures during the study. Third, the normal value of systolic BP is below 140 mmHg, and normal HR is between 60 /min and 100 /min in general. The average BP level was indeed higher than normal in our study participants, so we could not compare the lower BP groups results to normal BP patients in our study. As such, it remains unclear how potential changes in the acute autonomic dysfunction response to ICH might have influenced outcomes. Following this study, we aim to explore the influence of autonomic function on the entire ICH process and the pathogenesis between BP, HR, and ICH, using more dynamic monitoring data in the future.

## Conclusion

In summary, our study found that higher BP and HR levels are independently associated with a worse prognosis and lower survival rate among patients with ICH. The findings suggest that in patients with acute ICH, an initial higher BP and HR, which indicate autonomic dysfunction, may significantly impact the progression of the condition and the recovery process after the onset of ICH. These insights could guide the development of interventions and treatments that target BP and HR control to improve outcomes for patients with ICH.

## Data Availability

The data that support the findings of this study are available from the corresponding author upon reasonable request.
